# Supplementary material for: Knowledge, Attitudes and Practice Regarding Antibiotic Prescription by Medical Interns: A Qualitative Study in Spain
Source: Antibiotics (Basel). 2023 Feb 24;12(3):457. doi: 10.3390/antibiotics12030457 (PMC10044181; doi:10.3390/antibiotics12030457)
Supplement: Supplementary file 1 [file antibiotics-12-00457-s001.zip › Supplementary Data Table S1.pdf]

Table S1. Topics, and quotes by medical interns regarding factors that influence antibiotic use.		
1.1 Knowledge.	a. Own.	"*Cough without expectoration and, even though the patient doesn't have a temperature or anything, they send him home as a case of upper respiratory infection, which is viral, but they prescribe an antibiotic just in case...*" **FG1, M1**<br><br>"*(...) indeed, especially respiratory and urinary. Those are the cases in which they prescribe the most, without rhyme or reason.*" **FG2, M2**
	b. General population.	"*(...) and there's a lack of awareness among the public about just how dangerous excessive antibiotics are, and the fact that not everything calls for antibiotics.*" **FG3, W1.**
1.2 Healthcare burden	"*I can't devote all the time I'd like to, depending on the amount of people I've got waiting or the number of things I've still got to do on a duty shift. And one thing's for sure, it forces you to work faster and sometimes to make more mistakes.*" **FG5, W5.**<br><br>"*The time when the patient's admitted to the emergency ward. If it's late, like 5 or 6 in the morning, I think it's then when they're pneumonias or you just don't go beating your brains out (...) the first thing that occurs to me...*" **FG2, M2.**<br><br>"*I think it's more about apathy than lack of time. Because, well I mean, you can be short of time one day, but not for a whole month!*" **FG6, M3.**	
1.3 Inertia	"*(...) They're happy, their patients are happy, and there haven't been any problems, so they continue to trust that antibiotic.*" **FG3, W1.**<br><br>"*You learn from those who've gone before you and you learn from those around you, which is the tradition, which is what I like.*" **FG5, W5.**<br><br>"(...) *and it's like that, it's what you've seen in 60 people, you repeat it and hardly*	

	anyone studies it properly. So you've seen it or experienced it and you repeat that pattern a thousand times. You don't check to see whether this is right or I must check the guidelines. So yes, that's the way I think it works. It's just that, inertia." **FG6, M1.**
1.4 Pharmacological characteristics	"*And if it's in primary care, convenience of the dosage. For instance, in primary care when they give patients levofloxacin; because it's one tablet every twenty-four hours for three days and, if you explain to the patient and compare it with ten days of amoxicillin/clavulanic acid, it's obvious which he's going to want."* **FG1, W1.**<br><br>"*Between the fact that it has a fairly broad spectrum of action, has hardly any known side-effects, and everybody's super happy with augmentine. But quinolones have that tendon problem, they lower the rupture threshold... They always make you feel just a little more afraid."* **FG3, M1.**<br><br>"*Something else that really irritates me are packages of antibiotics that have more or fewer tablets than those shown in the guidelines, so in a case where you're going to cover 7 days with 20 tablets, they either take too many or too few, and they end up having tablets left over, which they throw away.*" **FG6, M1.**
1.5 Patient pressure	"*Oh patients are very demanding. You already know the patient's a very demanding person, you know him from other visits, and you don't feel like arguing with him. You give him the antibiotic and you know he's going to go away feeling easier, even though he doesn't really need it, and you avoid having an argument."* **FG6, W3.**<br><br>"*(...) This experience of 'I want you to give me antibiotics even though they're not indicated in my case' is far more common in the private sector where the patient thinks you're going to do what he tells you to do."* **FG2, W3.**
1.6 Complacency	"*Of course there's the complacency factor for the patient and the safety factor because*

towards the patient	*you say, -well I prescribed an antibiotic for him and I can forget about it-."* **FG6, M1.**
1.7 Complacency towards other physicians	"(...) *3 days ago, with the same disease and an identical patient, they told me to put him on this; now you tell me that I can't give it."* **FG2, M2.**<br><br>"*Yes, that's it. Children in emergencies who come with the antibiotics they've been prescribed by the private physician and GP even though you may not agree. Perhaps it's got a broader spectrum, and so on, and they don't normally take him off it."* **FG4, W1.**
1.8 Fear	"*Yes, but we often start empirically with the antibiotic before seeing the result. It's an entirely different situation where the patient later gets to see the result that may or may not confirm the initial suspicion. And if it isn't confirmed, well the treatment can be adjusted. But you always start by making sure to cover them."* **FG5, W5.**
1.9 Judgement of the attending physician	"*Yes I do, because they make me. I wouldn't prescribe half the antibiotics I prescribe, but if an attending physician tells me to prescribe, well I'm not going to say no."* **FG1, W2.**<br><br>"*(...) it makes no difference how they train us at university about not giving antibiotics, but you get to the hospital, and older doctors, who are supposed to have more experience (...), do it. So you're opinion doesn't count."* **FG3, M3**<br><br>"*Yes, when you don't know, you ask the attending physician and you prescribe what they tell you. You'll have time to check up afterwards, but you prescribe what they tell you and that's that."* **FG7, M1.**
1.10 External responsibility	"*If I had to pick one of them, I'd go for primary care."* **FG1, M1**<br><br>"*But let's spread the blame around a bit. It's also the fault of pharmacies that*

	dispense antibiotics without prescription." **FG4, W3.**<br><br>"*The antibiotics that they give to animals are also very important for the resistance that's around now."* **FG3, W1.**
