# Supplementary material for: Knowledge, Attitudes and Practice Regarding Antibiotic Prescription by Medical Interns: A Qualitative Study in Spain
Source: Antibiotics (Basel). 2023 Feb 24;12(3):457. doi: 10.3390/antibiotics12030457 (PMC10044181; doi:10.3390/antibiotics12030457)
Supplement: Supplementary file 1 [file antibiotics-12-00457-s001.zip › Supplementary Data Table S2.pdf]

Table S2. Needs perceived by medical interns in relation to the prescription of antibiotics.	
2.1 Complementary tests	"*Well I think without doubt that the signs are temperature, as well as leukocytosis and left axis deviation. With analytical tests.*" **FG5, W2.**
2.2 Training	*"Useful, yes!; accessible, no!... (...) I think a guideline should tell you what to prescribe this particular patient in these particular conditions, but a 90-page guideline isn't useful. In this regard, I think that (...) you study these guidelines at home but they'll never clear up a doubt."* **FG1, W1.**<br><br>"*We'd use antibiotics better if we had easier access to the information.*" **FG2, W1.**<br><br>"*Protocols, and everybody should follow the system laid down by each department.*" **FG5, W1**.<br><br>"*Social awareness, not only of health professionals, who I think hand out antibiotics like sweets, but also of the public. Because they're the first to demand them. If you don't give them to me, I'll go to another doctor tomorrow who* ***will*** *give them to me. And I feel that this is the main gap here.*" **FG3, W1.**<br><br>"*And who has to raise society's awareness. It could be the GP in his practice.*" **FG3, W3**
